# CT and MRI characteristic findings of sporadic renal hemangioblastoma: Two case reports

**DOI:** 10.1097/MD.0000000000024629

**Published:** 2021-02-12

**Authors:** Jie He, Nan Liu, Wangwang Liu, Wenli Zhou, Qiangfeng Wang, Hongjie Hu

**Affiliations:** a Department of Radiology; b Department of Critical Care Medicine; c Department of Pathology, Sir Run Run Shaw Hospital; d Department of Oncology, First Affiliated Hospital, Zhejiang University School of Medicine, Hangzhou, China.

**Keywords:** computed tomography, hemangioblastoma, magnetic resonance imaging, renal neoplasm, Von-Hippel-Lindau disease

## Abstract

**Rationale:**

Hemangioblastomas in the kidney are rare. Although a few cases of renal hemangioblastoma (RH) have been reported, the content of these articles mainly focused on clinical and pathological research, with minimal descriptions of radiologic findings. Moreover, there are no descriptions of magnetic resonance imaging (MRI) with enhancement of this condition. Herein, we report 2 cases of RH with computed tomography (CT) and MRI findings.

**Patient concerns:**

Two patients presented to our institution because of dull pain in the left abdomen, and a mass in the left kidney was found by ultrasound examination in each case. The patient had no special family history. Physical examination revealed no obvious tenderness or percussion pain in the renal and ureteral walking areas, and there was no obvious mass. Routine blood and urine tests were normal, and the serum tumor markers were negative. No obvious lesions were found on imaging of the other body parts.

**Diagnosis:**

Similar radiologic findings were observed in both cases and mimicked those of cavernous hemangiomas of the liver, including peripheral nodular enhancement in the corticomedullary phase, progressive centripetal enhancement in the nephrographic and delayed phases, and occasional complete “filling in” in the delayed phase. We made a radiologic diagnosis of renal clear cell carcinoma for patient 1 and suspected renal clear cell carcinoma for patient 2, but the pathological results showed RH.

**Interventions:**

Given the suspicion of renal cell carcinoma, both patients underwent partial nephrectomy.

**Outcome:**

The recovery of the two patients was uneventful, and there was no evidence of local recurrence or metastasis many years after surgery.

**Lessons:**

RH is a rare benign tumor that can be easily misdiagnosed as clear cell carcinoma. Characteristic CT and MRI manifestations may improve preoperative diagnostic accuracy to avoid surgery or indicate nephron-sparing surgery.

## 1 Introduction

Hemangioblastomas are vascular tumors that often occur in the central nervous system (CNS), especially in the cerebellum. Most cases are sporadic, and about 20% to 38% of patients also have Von-Hippel-Lindau (VHL) disease, which is an autosomal dominant genetic condition with an incidence of 1/27,300 to 1/45,000.^[1–3]^ The VHL gene is located on chromosome 3p25–26 and is an important tumor suppressor gene that contains three exons.^[4,5]^ The VHL gene is transcribed to a 4.5-kb-long mRNA that encodes a VHL protein (PVHL) containing 213 amino acids. Loss, mutation, or methylation inactivation of the *VHL* gene disturbs PVHL synthesis, which is an important molecular basis for VHL disease. VHL-related tumors include hemangioblastomas that are usually located in the CNS, fewer renal and pancreatic cystic tumors, neuroendocrine tumors of the pancreas, renal clear cell carcinoma, endolymphatic sac tumors, pheochromocytomas, and paragangliomas.^[2,6]^

Beyond the CNS, hemangioblastomas can also occur in the peripheral nervous system,^[7,8]^ retroperitoneum,^[9,10]^ pelvic cavity,^[11]^ soft tissue,^[12]^ bone,^[13]^ adrenal glands,^[14]^ lungs, and liver.^[15]^ The PubMed database was searched for literature using the keywords “hemangioblastoma” and “kidney or renal.” After screening all articles in the database, the first case report of renal hemangioblastoma (RH) was reported by Nonaka et al in 2007.^[16]^ RH is rare, and no more than 30 cases have been reported, but the content of these articles mainly focused on clinical and pathological research with limited descriptions of radiologic findings.^[17–27]^ Moreover, there are no descriptions of enhanced MRI findings of RHs. To the best of our knowledge, this is the first report of enhanced MRI findings.

We report 2 cases of patients with RH confirmed by surgery and pathology with complete clinical, pathological, and imaging data collected in our hospital. Neither patient had VHL disease, and was diagnosed with sporadic RH.

## 2 Methods

### 2.1 Computed tomography (CT) examination

A 64-slice spiral contrast-enhanced (CE) abdominal CT (Somatom Definition AS; Siemens, Erlangen, Germany) was used for both patients. The CT scanning parameters were as follows: reconstruction thickness of 2 mm, reconstruction of increment, pitch of 1.25, tube voltage of 120 kV, and tube current of 170 to 360 mAs using automatic tube current modulation technology. CE abdominal CT scans were performed with the patient in a supine position and scanned from the xiphoid process to the anterior superior iliac level. After the plain scan, the patient received intravenous nonionic iodinated contrast agent iohexol (320 mgI/mL) as a 75-mL bolus at a rate of 3.0 mL/s, followed by 50 mL of normal saline. Corticomedullary and nephrographic phase images were obtained 30 and 60 seconds after contrast agent injection, respectively.

### 2.2 MRI examination

MRI examinations were performed using a Signa HDxt 3.0T magnetic resonance (MR) system (GE Healthcare, Chicago, IL), with the use of the body coil as a transmitter and an 8-channel phased-array coil as the receiver. The detailed examination protocols are summarized in Table 1. T1-weighted fat-suppressed dynamic CE MR images were acquired using the liver acquisition with volume acceleration-extended volume sequence before and 40, 100, and 160 seconds after intravenous administration of 0.1 mmol/kg of gadolinium (Magnevist; Bayer HealthCare Pharmaceuticals, Leverkusen, Germany). Coronal T1-weighted CE images were acquired, and the patient was injected with 20 mL normal saline.

**Table 1:** MRI acquisition parameters.

Parameter	Fat-suppressed T2-weighted	DWI	T1-weighted	Dynamic	Late postcontrast
Imaging plane	Axial	Axial	Axial	Axial	Coronal
Fat suppression	Yes	Yes	No	Yes	Yes
TR/TE, ms	6316/87	5714/59	290/2.4 in-phase 290/5.8 opposed phase	2.59/1.19	3.44/15
FA, °	90	90	80	11	12
Slice thickness, mm	6	6	6	4.4	2.8
Slice gap, mm	7	7	7	2.2	1.4
Number of slices	22	38	44	368	96
Delay, s	/	/	/	0,40,100, 160	190
b-values, s/mm^2^	/	0 and 600	/	/	/

DWI = diffusion-weighted imaging, FA = flip angle, TE = echo time, TR = repetition time.

## 3 Case presentation

### 3.1 Case 1

In November 2015, a 45-year-old male patient presented to our institution with a dull pain in the left abdomen that had been present for 2 weeks. The patient had never undergone surgery and had an unremarkable family history. The respiratory frequency of the patient was 18 times per minute, with a body temperature of 36.7°, pulse rate of 76 beats per minute, and blood pressure of 110/76 mm Hg. Physical examination revealed no obvious tenderness or percussion pain in the renal or ureteral walking area, and there was no palpable mass. Routine blood and urine tests were normal, and serum carbohydrate antigen 199, carbohydrate antigen 125, alpha-fetoprotein, carcinoembryonic antigen, and prostate-specific antigen were negative. No obvious lesions were found on chest X-ray, head CT, or ultrasonography of the liver, gallbladder, pancreas, and spleen.

**Figure 1:**
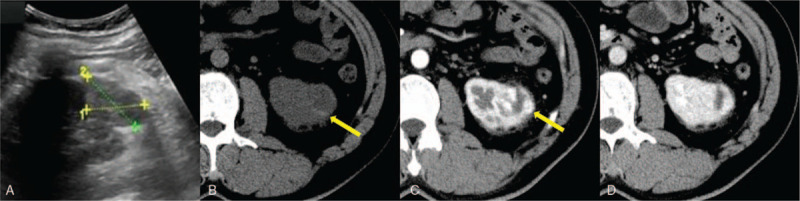
A 45-year-old man with a round mass in the left kidney diagnosed as hemangioblastoma. (A) Ultrasonography revealed a 37 × 42 mm heterogeneous echo mass inside the lower pole of the left kidney that had an irregular shape and a distinct margin. (B) Axial CT images of the precontrast phase of the abdomen showed a round soft tissue mass in the lower pole of the left kidney, and a small high-density shadow could be seen in the periphery of the mass (yellow arrow), which suggested a hemorrhage. (C) In axial CT images of the corticomedullary phase, the mass showed peripheral nodular enhancement (yellow arrow), similar to that of the abdominal aorta at the same level. (D) Axial CT images of the nephrographic phase showed slight progressive centripetal enhancement.

Ultrasonography (Fig. 1A) revealed a 37 × 42 mm heterogeneous echo mass inside the lower pole of the left kidney, which had an irregular shape and distinct margin. There was some echo of the blood flow inside the patient. CT and MRI findings are shown in Figure 1b--d and 2 A-G, respectively. A round, soft tissue density or signal mass was observed inside the lower pole of the left kidney. In the periphery of the mass, some small high-density areas in CT images of the precontrast phase and hypointensities in fat-suppressed T2-weighted images and diffusion-weighted images suggested hemorrhage. After contrast agent injection, the mass showed peripheral nodular enhancement in both CT and MR images of the corticomedullary phase and progressive centripetal enhancement on CT and MR images of the posterior enhanced phase. In the center of the mass, hyperintensity in fat-suppressed T2-weighted images and diffusion-weighted images that were not enhanced suggested necrosis. We made a radiologic diagnosis of renal clear cell carcinoma because the mass showed a rich blood supply with hemorrhage and necrosis.

**Figure 2:**
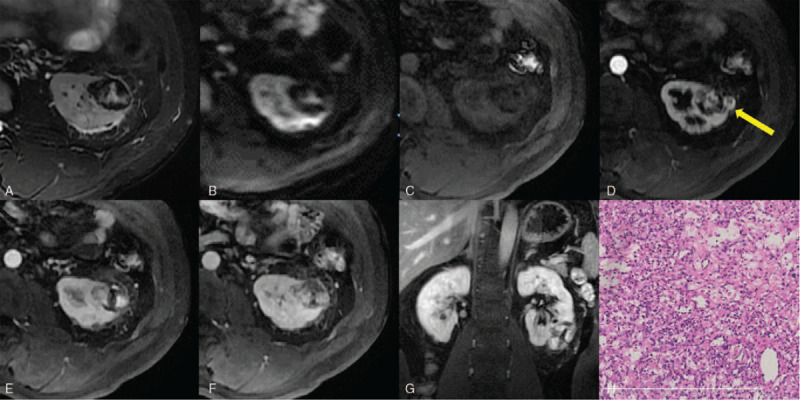
(A) Axial fat-suppressed, T2-weighted image and (B) diffusion-weighted image showing hypointensity in the periphery of the mass suggesting hemorrhage, and hyperintensity in the center of the mass indicative of necrosis. (C) In axial, precontrast, fat-suppressed, T1-weighted images, the mass appeared heterogeneously hypointense compared with the renal parenchyma. (D) Axial, corticomedullary phase, fat-suppressed, gadolinium-enhanced, T1-weighted images showed peripheral nodular enhancement (yellow arrow). (E) Axial nephrographic phase, (F) axial delayed phase and (G) coronal delayed phase fat-suppressed, gadolinium-enhanced, T1-weighted images showed progressive centripetal enhancement. An area in the center of the mass was not enhanced. (H) Histological examination demonstrated an RH. The tumor was mainly composed of a rich vascular network interspersed with polygonal cells, HE × 100.

Given the suspicion of renal cell carcinoma (RCC), the patient underwent laparoscopic right partial nephrectomy. The pathological results revealed an RH (Fig. 2H). Immunohistochemical results were cytokeratin (CK)-pan (partially +), CD31 (-), CD34 (-), inhibin alpha (+), neuron-specific enolase (NSE) (partially +), vimentin (+), and Ki-67 (1%+). The patient's recovery was uneventful, and there was no evidence of local recurrence or metastasis 5 years after surgery.

### 3.2 Case 2

In December 2017, a 42-year-old female patient presented to our institution with a mass in the left kidney found during an ultrasound examination in a local hospital. The patient had a history of cesarean section 16 years earlier and had no special family history. Her respiratory frequency was 21 beats per minute, with a body temperature of 36.5°C, pulse rate of 78 beats per minute, and blood pressure of 100/58 mm Hg. Physical examination revealed no obvious tenderness or percussion pain in the renal or ureteral walking area, and there was no obvious mass. Routine blood and urine tests were normal, and serum carbohydrate antigen 199, carbohydrate antigen 125, alpha-fetoprotein, carcinoembryonic antigen, and prostate-specific antigen were negative. No obvious lesions were found on chest radiography or ultrasonography of the liver, gallbladder, pancreas, or spleen.

**Figure 3:**
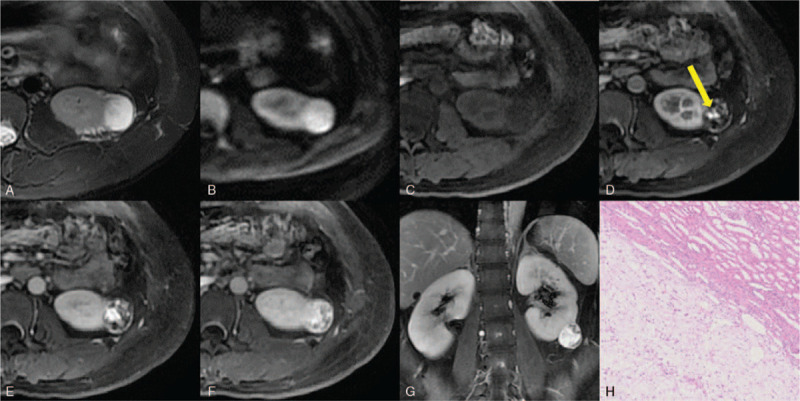
A 42-year-old woman with a 29 × 26 × 28 mm round mass in the left kidney diagnosed as hemangioblastoma. (A) Axial, fat-suppressed, T2-weighted images and (B) diffusion-weighted images showed significant hyperintensity of the mass. (C) In axial precontrast, fat-suppressed, T1-weighted images, the mass appeared hypointense compared with the renal parenchyma. (D) Axial corticomedullary phase fat-suppressed, gadolinium-enhanced, T1-weighted images showed peripheral nodular enhancement (yellow arrow). (E) Axial nephrographic phase, (F) axial delayed phase, and (G) coronal delayed phase fat-suppressed, gadolinium-enhanced, T1-weighted images showed progressive centripetal enhancement and almost complete “filling in” in the delayed phase. (H) The tumor was surrounded by a fibrous capsule and had a well-demarcated border against the surrounding renal parenchyma, HE × 100.

The MRI findings are shown in Figure 3A–G. A 29 × 26 × 28 mm round soft tissue signal mass was observed inside the left kidney. The mass showed significant hyperintensity in fat-suppressed T2-weighted images and diffusion-weighted images, which could be described as the “light bulb sign.” After contrast agent injection, the mass exhibited peripheral nodular enhancement in MR images of the corticomedullary phase and progressive centripetal enhancement in MR images of the nephrographic and delayed phases, with almost complete “filling in” in the delayed phase. The radiological diagnosis was suspected renal clear cell carcinoma because the renal mass showed unique findings that we had not previously seen in renal masses.

Given the suspicion of RCC, the patient underwent da Vinci robot-assisted left partial nephrectomy. The pathological results showed RH, and the tumor was surrounded by a fibrous capsule (Fig. 3H). Immunohistochemical results were CK-pan (-), CD31 (-), CD34 (-), inhibin alpha (+), NSE (+), PAX-8 (+), RCC (-), and S-100 (+). At follow-up of 3 years after surgery, the patient was in good health without recurrence.

## 4 Discussion

The latest 2016 World Health Organization classification of tumors of the urinary system and male genital organs now includes RH as a mesenchymal tumor, which is similar to CNS hemangioblastoma.^[28]^ However, a correlation between VHL syndrome and VHL gene mutation has not been reported, and the biological behavior of the tumor is benign. RH is a rare, slow-growing, benign renal tumor. It usually affects middle-aged and elderly patients, but can also occur in younger patients.^[18,20]^ Compared with women, men are 1.5 to 2 times more likely to develop RH.^[29]^ In sporadic cases such as those described here, there is no VHL-related disease and no associated family history of VHL disease in the patient (so-called “isolated” RH).^[26]^

The gross features of RH include a predominantly solid tumor rich in capillaries with a well-demarcated border from the surrounding renal parenchyma, which is consistent with what we observed in the surgical specimens. Microscopically, RH is a morphologically distinctive vascular neoplasm with rich capillary networks and lipid-rich stromal cells.^[18,20,29]^ On the basis of the stromal cell components, they could be divided into 3 types: capillary-dominated, interstitial cell-dominated, and classic type (intermediate between the other 2 types). Unfortunately, although the stromal cells of RH appear normal, they may show obvious nuclear pleomorphism, similar to malignant tumors. Therefore, RH can be easily misdiagnosed as RCC or other malignant tumors.^[29]^

Because it is difficult to distinguish RH from renal clear cell carcinoma by routine hematoxylin and eosin (HE) staining alone, immunohistochemical examination is helpful for differential diagnosis. Studies have shown that labeling with inhibin alpha, S-100, and CD10 helps distinguish RH from renal clear cell carcinoma. Inhibin alpha and S-100 are usually positive in RH and negative in renal clear cell carcinoma and contrast; CD10 is usually positive in renal clear cell carcinoma and negative in RH.^[30–33]^ Our results are consistent with the results of the above studies.

CT and MRI are very useful examination methods that have been widely used in the diagnosis and differential diagnosis of diseases in various systems. Both have gained increased acceptance for the accurate diagnosis and differential diagnosis of renal tumors.^[34–37]^

Most hemangioblastomas are in the cerebellum and can be divided into 3 types: solid, solid-cystic, or predominantly cystic with small mural nodules.^[38,39]^ The most common and typical radiologic findings correspond to the last type and show a markedly enhanced small mural nodule attached to a large unenhanced cyst wall.^[40,41]^ There is limited literature describing the imaging findings of RH, but they report RH as a solid, heterogeneously enhanced mass, which is consistent with our cases.^[17,22,23]^ However, they do not correspond with the most common and typical radiological findings of hemangioblastoma in the cerebellum. This discrepancy may be related to the different tumor growth environments.

In addition to solid tumors, the CT and MRI findings of our 2 patients were similar to those of cavernous hemangiomas of the liver, including peripheral nodular enhancement in the corticomedullary phase, progressive centripetal enhancement in the nephrographic and delayed phases, and sometimes complete “filling in” in the delayed phase.^[42,43]^ Other renal tumors do not exhibit these enhancement patterns, which may be unique to RH. At present, these findings have not been reported in the literature. It may be that others have not noticed this feature, or they may not have performed CE multiphase scanning with CT and MRI. T2WI also helps in the differential diagnosis. The high signal intensity on T2WI indicated slow blood flow in the neoplastic vascular channel, which is of great significance to angiogenic tumors. The first case was less typical due to bleeding, but the second case was similar to cavernous hemangioma of the liver, showing a significantly high signal intensity known as the “light bulb sign.”^[44]^

Because RHs are indolent neoplasms, asymptomatic tumors may be managed with observation. Gross total resection is the most suitable treatment if an intervention is required.^[45]^ If a correct preoperative diagnosis was made, these 2 patients may have received different treatments. Unfortunately, RH can easily be misdiagnosed as renal clear cell carcinoma, the most common malignant tumor of the kidney, which can lead to overtreatment. Characteristic CT and MRI manifestations may help guide the preoperative diagnosis. At the same time, once RH is surgically confirmed, a comprehensive examination should be performed to determine if the tumor is associated with VHL disease.

In conclusion, RH is a rare benign tumor that can be easily misdiagnosed as clear cell carcinoma. Characteristic manifestations on CT and MRI may help us make a preoperative diagnosis to avoid surgery or indicate nephron-sparing surgery. This highlights the importance of accurate preoperative radiologic diagnoses.

## Author contributions

**Data curation:** Jie He, Wangwang Liu.

**Funding acquisition:** Qiangfeng Wang.

**Methodology:** Wenli Zhou, Hongjie Hu.

**Writing – original draft:** Jie He, Nan Liu, Hongjie Hu.

**Writing – review & editing:** Hongjie Hu.

## Abbreviations

Abbreviations: CK = cytokeratin, CNS = central nervous system, CT = computed tomography, DWI = diffusion-weighted imaging, FA = flip angle, HE = hematoxylin and eosin, MRI = magnetic resonance imaging, NSE = neuron-specific enolase, PVHL = VHL protein, RCC = renal cell carcinoma, RH = renal hemangioblastoma, TE = echo time, TR = repetition time, VHL = Von-Hippel-Lindau.
